# Autistic adults form first impressions from voices in similar ways to non‐autistic adults

**DOI:** 10.1111/bjop.70006

**Published:** 2025-07-03

**Authors:** Ceci Qing Cai, Rong Ma, Terry Hin Ng, Sarah J. White, Nadine Lavan

**Affiliations:** ^1^ Institute of Cognitive Neuroscience University College London London UK; ^2^ Centre for Brain and Behaviour, School of Biological and Behavioural Sciences Queen Mary University of London London UK

**Keywords:** age, autism, first impressions, trustworthiness, voice perception

## Abstract

In everyday life, listeners spontaneously and rapidly form first impressions from others' voices. Previous research shows that, compared to non‐autistic people, autistic people show similarities and differences in how they evaluate others based on their faces. However, it remains unclear whether autistic people form first impressions from voices in the same way as non‐autistic people. We asked both autistic and non‐autistic listeners to rate an inferred characteristic (trustworthiness) and an apparent characteristic (age) from voice recordings to establish how they form first impressions from voices. Non‐autistic and autistic listeners formed first impressions for age and trustworthiness in similar ways. Specifically, both groups showed comparable overall ratings of age and trustworthiness of voices. Further, both autistic and non‐autistic listeners required similar amounts of information to form an impression. Finally, when comparing trait impressions within‐ and across‐groups, we again found no systematic evidence of impression formation differing between autistic and non‐autistic people. These findings indicate that first impression formation is potentially a relative social strength in autism. This suggests that the social challenges encountered by autistic people may be confined to specific areas of social perception rather than being universally pervasive.

## BACKGROUND

When we first hear a new voice, we rarely perceive this sound as simply ‘a voice’ and no more—instead, we infer a wealth of socially relevant information from voices. For example, listeners can instantly form a complex first impression of what a person might be like from hearing their voice for only a fraction of a second (Lavan & McGettigan, 2023). While some of these first impressions are known to be accurate to an extent (cf. impressions of sex, age, weight, height, etc.; Sorokowski et al., 2023), others, for example, judging another's personality characteristics from their voice, have little grounding in reality (Todorov et al., 2015). While not necessarily accurate, these impressions have real‐world impact as they affect people's behaviour and decision‐making; for example, first impressions from candidates' voices can influence voting behaviour (Klofstad, 2017; Tigue et al., 2012).

While these impressions are multifaceted, such that we build a picture of a person's perceived gender, age, social status and personality, these impressions are underpinned by a low‐dimensional ‘trait space’ that primarily focuses on personality traits, organized around a small number of basic dimensions including trustworthiness, dominance and attractiveness (McAleer et al., 2014; Oosterhof & Todorov, 2008). This kind of impression formation is known to be rapid (Lavan, 2023; Lavan et al., 2024; McAleer et al., 2014; Mileva & Lavan, 2023). Less than a second of exposure to a voice is enough to form a first impression. Many socially relevant first impressions based on inferred characteristics (e.g. degree of education, ‘posh‐ness’, trustworthiness) can be formed from around 400–800 ms of exposure, even though such characteristics are not directly conveyed by the voice (e.g. a person may or may not be trustworthy regardless of how they sound). In contrast, impressions of apparent characteristics (e.g. sex, age, health), which are to some extent directly perceivable from the voice (e.g. older individuals tend to sound older due to acoustic changes over time), can be formed extremely quickly, from only 25 ms of exposure (Lavan, 2023; Lavan & Sutherland, 2024). Almost all impressions we form from voices are to some degree shared across listeners (Lavan et al., 2024; McAleer et al., 2014; Mileva & Lavan, 2023), such that people on average agree whether a voice sounds, for example, more or less trustworthy. This shared nature of impressions can be explained by some impressions being either accurate or influenced by (shared) social stereotypes. Crucially, however, not all aspects of first impressions are completely shared among listeners; some recent work shows that there is also a role for individual differences and idiosyncrasy in impression formation (Lavan & Sutherland, 2024). For example, while one person may consistently perceive lower‐pitched voices as sounding more trustworthy, another person might associate higher‐pitched voices with higher trustworthiness.

Finding that there are individual differences in impression formation across the population opens up the question of whether there are different groups of listeners who may consistently differ from one another in terms of how they form social impressions. One such group is autistic people, who are characterized by persistent difficulties in social communication and interaction (DMS‐5‐TR; American Psychiatric Association, 2022). Differences in the processing and understanding of social information between autistic and non‐autistic people have been observed across facial expressions, body movements and gaze patterns (Cook, 2016; Hamilton, 2016; Trevisan et al., 2018). However, much less attention has been given to how autistic people process auditory cues, such as voices, despite the crucial role of vocal information in everyday social interactions.

The small existing literature on the perception of auditory cues in autism addresses both lower‐level and higher‐order auditory stimuli. When considering low‐level pitch perception from voice stimuli, findings from the autism literature have been mixed. Some studies suggest that autistic people struggle to judge vocal pitch differences (Schelinski & von Kriegstein, 2019), particularly when recognizing and discriminating unfamiliar from familiar voices (Schelinski et al., 2017). [Corrections added on 29 July 2025, after first online publication: the citation of Järvinen‐Pasley & Heaton (2007) reference has been changed to Schelinski & von Kriegstein, 2019.] This aligns with findings that autistic people may struggle more generally with tasks requiring fine auditory discrimination (Kargas et al., 2015). However, some research indicates that autistic people may be more sensitive to pitch changes and demonstrate superior performance compared to non‐autistic people in lower‐level pitch processing (Bonnel et al., 2003; Järvinen‐Pasley & Heaton, 2007; Jiang et al., 2015; Stanutz et al., 2014), highlighting variability in their auditory processing abilities (Jones et al., 2009). [Corrections added on 29 July 2025, after first online publication: the citation of Järvinen‐Pasley & Heaton (2007) reference has been added to this version.] Regarding voice processing, one further study reports contradictory results where autistic people perform similarly to non‐autistic people in vocal identity recognition and are superior to non‐autistic people in discriminating between familiar or unfamiliar voices (Lin et al., 2015).

Similarly, contrasting findings have been reported for higher‐level vocal emotion processing. While one study identified difficulties in perceiving both vocal emotion and pitch in autism, suggesting that lower‐level vocal perception differences may contribute to autistic challenges in vocal emotion recognition (Schelinski & von Kriegstein, 2019), a recent review indicates that accuracy in recognizing emotion in auditory cues does not appear to be directly related to pitch perception in autism. In contrast, non‐autistic people appear to rely heavily on pitch for vocal emotion recognition (see Leung et al., 2022). Indeed, some studies show that autistic people are comparable to their non‐autistic peers in accurately recognizing basic emotions—such as happiness, anger and fear—not only from both verbal and non‐verbal emotional vocalizations (Jones et al., 2011) but also from speech prosody and song (Leung et al., 2023), particularly when identifying the emotion context from speech produced by autistic speakers rather than non‐autistic speakers (Hubbard et al., 2017). Other, arguably more subtle, differences in vocal emotion perception arise between autistic and non‐autistic people when understanding more complex socio‐emotional meanings: while autistic people can differentiate between genuine and posed laughter, they tend to perceive posed laughter to be more similar to genuine laughter than non‐autistic people (Cai, White, et al., 2024); they also face challenges detecting complex emotion, such as sarcasm, in speech prosodic cues (Diehl et al., 2015) and show reduced differentiation of emotion in speech when the acoustic variability and volatility increase (Duville et al., 2024).

These mixed findings at both lower and higher levels of voice processing raise questions about how trait perception from voices in autism differs; specifically, whether they quickly form a shared impression of vocal traits with other autistic and non‐autistic people or whether differences arise due to their challenges in social perception. Only very limited research has examined trait perception from vocal cues in autism. Autistic people seem to show abilities comparable to non‐autistic people in judging apparent traits from voices, such as identifying gender (Groen et al., 2008; Lin et al., 2015) and estimating age (Clopper et al., 2012). However, there have been no studies of social trait perception from voices in autism. Studies of trait perception from faces broadly show that autistic people judge social traits similarly to non‐autistic people in terms of trustworthiness and social status (White et al., 2006). An online study with a larger sample likewise found that autistic people make high‐level social trait judgements from faces similarly to non‐autistic people on dimensions such as warmth, competence, femininity and youth (Cao et al., 2023). There are, however, some specific differences in judging others, such as attractiveness for same‐gender faces, and autistic people exhibit lower within‐group agreement in their ratings, along with a tendency toward more positive evaluations (Cao et al., 2023). It remains an open question, therefore, whether social impressions from voices are formed in the same way in autistic and non‐autistic people.

To date, no studies have investigated how autistic people form split‐second impressions of others from voices. In the current study, we investigated whether autistic people differ from non‐autistic people in how they form first impressions from voices across varying exposure durations. We therefore asked autistic and non‐autistic participants to complete a trait perception task, in which participants rated 100 voices either for how trustworthy or how old they sounded. Participants rated the voice clips at 50, 200 and 800 ms of exposure (see Lavan, 2023; Mileva & Lavan, 2023) in order to examine the degree of exposure needed for different listeners to form an impression of a voice. We included trustworthiness vs. age to contrast an inferred characteristic with a more apparent characteristic to examine the specificity of any differences to inferred characteristics. We predicted that there would be no differences in mean ratings for age and trustworthiness between autistic and non‐autistic adults for longer exposures. However, under the greater processing demands of short durations, we predicted that autistic people would struggle to form consistent impressions of inferred traits. We also predicted that impressions of age would be formed more quickly than impressions of trustworthiness in both groups (Lavan, 2023).

## METHODS

### Participants

Forty autistic adults and 40 non‐autistic adults participated in this study. All participants were fluent in English, right‐handed and had no speech or hearing difficulties. Non‐autistic participants were recruited from local participant databases. Autistic participants were recruited through autism communities and databases across the UK and had previously received an official diagnosis from a qualified clinician and provided diagnostic reports prior to testing. Fourteen of the 80 participants (10 autistic; 4 non‐autistic) chose to take part online. These participants had participated in previous in‐person studies, with existing background data already available in our database. They were offered the option to take part online to maximize participation as they were not attending in‐lab sessions on the same day. This approach helped us meet recruitment targets while maintaining sample size and consistency with prior studies (Lavan, 2023; Mileva & Lavan, 2023). Informed written consent was obtained from all participants prior to testing, and the study received approval from the university research ethics committee.

The groups were comparable on sex, *χ* ^2^(1) = 0.800, *p* = .371, age, *t*(78) = 0.162, *p* = .872, and verbal performance, *t*(78) = 1.555, *p* = .124, performance, *t*(78) = 0.596, *p* = .553 and full‐scale IQ, *t*(78) = 1.227, *p* = .224 (Wechsler, 2008), but differed on the AQ (Baron‐Cohen et al., 2001), *t*(77) = 12.591, *p* < .001. Full details of the groups are presented in Table 1.

**TABLE 1:** Demographic details of the groups.

	Non‐autistic	Autism
*N* (male:female)	40 (18:22)	40 (22:18)
Age (years)	33.03 (11.06)	33.45 (12.42)
Verbal IQ	108.15 (13.29)	112.98 (14.45)
Performance IQ	114.73 (15.74)	116.75 (14.62)
Full Scale IQ	113.55 (15.66)	117.93 (16.24)
AQ	17.40 (7.23)	36.39 (6.12)

*Note*: Values are given as mean (standard deviation). One autistic participant did not complete the AQ questionnaire.

Abbreviation: AQ, autism‐spectrum quotient.

### Materials

The voice stimuli used in the current study are from the same stimuli set used in previous studies (Lavan, 2023; Mileva & Lavan, 2023). Specifically, the stimuli were sampled from the Saarbrücken Voice Database (Pützer & Barry, n.d.). One hundred voice recordings of young adults (50 male, 50 female) were used. Each speaker produced the vowel /a/ in a sustained manner at a comfortable pitch. All recordings were then trimmed into different exposure durations. In this study, we selected exposure durations of 50, 200 and 800 ms for the voice recordings. The shortest exposure duration of 50 ms was selected because voice recordings that are shorter than 50 ms are less likely to be perceived as human voices (see Mileva & Lavan, 2023). The 800 ms duration was selected as it has been shown in previous work that this amount of exposure is sufficient to form first impressions for a wide range of person characteristics from voices (see Lavan, 2023).

### Task and procedure

**FIGURE 1:**
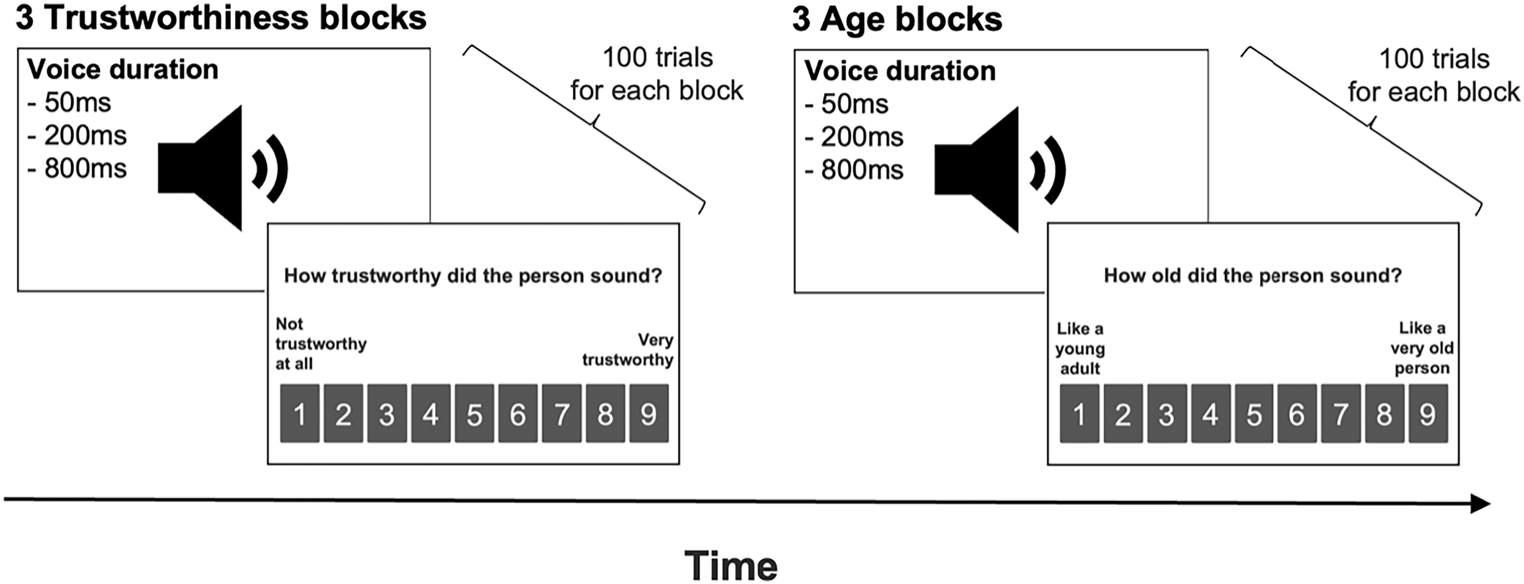
Illustration of the experimental task. The trustworthiness blocks were presented before the age blocks, and the three exposure duration blocks were randomly presented within each characteristic. On each trial, participants were presented with a voice, followed by a 9‐point rating scale for a specific characteristic of that voice.

The experimental design was adapted from the paradigm by Mileva and Lavan (2023) and Lavan (2023) and was created and hosted using Gorilla Experiment Builder (www.gorilla.sc; Anwyl‐Irvine et al., 2020). In the current study, participants rated trustworthiness and age as an inferred and apparent characteristic. Participants were presented with voice clips and were then instructed to judge these voices using a 9‐point scale for age (1—‘like a young adult’; 9—‘like a very old person’) and trustworthiness (1—‘not trustworthy at all’; 9—‘very trustworthy’). For both characteristics, each participant completed three blocks of ratings for the different exposure durations (50, 200 and 800 ms) with 100 voice clips each. The order of the blocks was randomized, with stimuli also randomly presented within each block. The trustworthiness blocks were presented before the age blocks to ensure that participants gave trustworthiness ratings based on their intrinsic perception of the voice stimuli, rather than through a second evaluation. Thus, participants in total completed 600 ratings (100 voice stimuli × 3 exposure durations × 2 characteristics), across six blocks (Figure 1).

Attention checks were embedded within each block to ensure participants maintained focus on the task. Specifically, nine vigilance trials were randomly presented within each block, instructing participants to select a specific number, such as ‘Please select 9’. To minimize fatigue and reduce carryover effects, participants were given a 30‐s break between blocks and a 60‐s break after completing three blocks of ratings.

The experimental design was identical for both in‐lab and online data collection, with the only difference being that the online version did not include the AQ questionnaire or demographic information sheet as this information had already been collected from online participants. In contrast, the in‐lab version included these components and an additional 1‐h IQ test. Participants first read the information sheet and completed the consent form. To minimize distractions and account for potential sensory sensitivities, only one participant was tested in the room at a time during in‐lab testing. All participants, whether they participated online or in‐lab, were instructed to (1) set their browser to full screen and (2) wear headphones and adjust the volume to a comfortable level. Participants must confirm these actions by ticking a checkbox before proceeding to the sound check. During the sound check, participants were presented with an audio clip of the word ‘chocolate’, which they could play as many times as needed. They were required to type the word correctly into a text box before progressing to the actual task. This procedure ensured that all participants completed the task in a quiet environment where they could hear the stimuli clearly. After a short practice (2 trials, 2 vigilance trials), participants proceeded with the voice stimuli ratings task, which lasted approximately 40 min. The experimenter remained with the participant during the sound check and practice trials to provide instructions and ensure their comfort but left the room once the main experiment began. All participants were compensated £9 per hour for their participation.

## RESULTS

We excluded data for individual participants on a block‐by‐block basis. In total, we excluded 17 blocks of ratings from 13 participants (autistic, *n* = 9); 6 blocks (trust, *n* = 2) from 4 participants (autistic, *n* = 3) were excluded for failing more than 20% of vigilance trials in one block (9 vigilance trials in total per block). Additionally, 11 blocks (trust, *n* = 11) were excluded from 9 participants (autistic, *n* = 7) for using a single rating response for more than 80% of the trials in one block (e.g. selecting ‘5’ for all experimental trials). Notably, the results of our study remained consistent regardless of whether those data were included or excluded in our analysis.

### Do autistic and non‐autistic listeners differ in how they rate voices on average?

**FIGURE 2:**
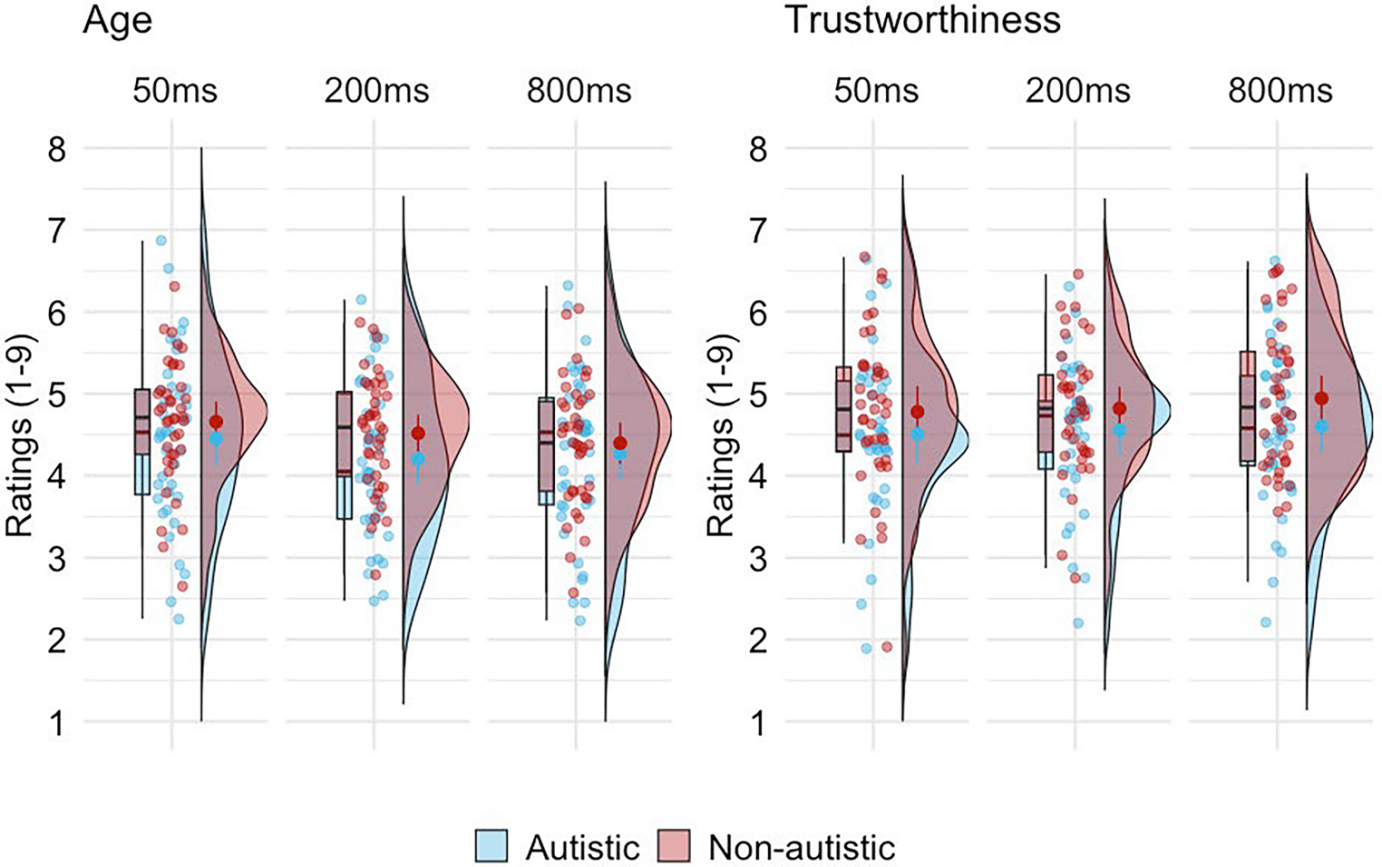
Mean ratings of each characteristic at each exposure duration, separately for age (left) and trustworthiness (right). Boxplot and raincloud plots represent the distribution of the ratings. Each light dot represents a participant's average rating of each characteristic at each exposure duration. Each dark dot with a line represents the mean ± 1 *SE*.

To investigate whether autistic and non‐autistic listeners differed in how they on average perceived person characteristics from voices, we first calculated the mean ratings of trustworthiness and age for each exposure duration in both autistic and non‐autistic groups (Figure 2). A Bayesian two‐way repeated measures ANOVA (2 groups × 3 durations) was conducted separately for age and trustworthiness ratings. We used Bayesian ANOVA because it offers interpretive advantages over frequentist approaches, particularly in contexts like our study where both the presence and absence of effects are theoretically meaningful (Jarosz & Wiley, 2014; van den Bergh et al., 2020). Bayes factors quantify the relative predictive success of competing models, enabling direct comparison of evidence for the null and alternative hypotheses and distinguishing the true absence of effects from inconclusive results (BF ≈ 1). In addition, Bayesian inference supports robust conclusions and is not compromised by issues such as optional stopping or sample size dependence. Throughout the Results, Bayes factors are reported as BF_01_, where values greater than 1 indicate evidence in favour of the null model and values less than 1 indicate evidence in favour of the alternative model (Jarosz & Wiley, 2014; van den Bergh et al., 2020). All analyses were conducted using JASP (version 0.19.3; JASP Team, 2025) with default priors, following the recommendations outlined in van den Bergh et al. (2020).

For age ratings, model comparison favoured the duration‐only model, BF_01_ = 1.000, *P*(*M*|data) = 0.548, indicating that this was the most plausible model given the data. This model was compared against a set of alternative models, including the null model, the full model and intermediate models (see Table 2). The Bayes factor for the duration effect (BF_01_ = 0.011) provided very strong evidence in favour of an effect of duration. In contrast, the Bayes factors for the group effect (BF_01_ = 1.853) and the interaction between duration and group (BF_01_ = 3.133) provided moderate and moderate to strong evidence, respectively, in favour of the null model. This means that while stimulus duration affected age ratings, group membership did not affect ratings, such that neither adding a group effect nor an interaction between group and duration effect improved the model's explanatory power compared to the simpler duration‐only model.

**TABLE 2:** Model comparison for all models under consideration for age ratings.

Models	*P*(*M*)	*P*(*M*|data)	BF_M_	BF_01_	Error %
Duration	0.200	0.548	4.857	1.000	
Duration + Group	0.200	0.371	2.356	1.479	3.013
Duration + Group + Duration × Group	0.200	0.074	0.319	7.421	2.410
Null model	0.200	0.004	0.017	128.191	0.583
Group	0.200	0.003	0.011	196.648	1.806

*Note*: All models include subject and random slopes for all repeated measures factors.

To further examine the effect of duration on age ratings, we conducted Bayesian post hoc analyses. Comparisons showed very strong evidence for differences between 50 and 200 ms (BF_01_ = 0.042) and between 50 and 800 ms (BF_01_ = 0.012), indicating that age ratings were substantially higher for the shortest exposure duration, irrespective of whether listeners were autistic or not. In contrast, the comparison between 200 and 800 ms (BF_01_ = 7.830) provided moderate to strong evidence in favour of the null, suggesting no meaningful difference between these two longer exposure durations.

For trustworthiness ratings, model comparison favoured the null model, BF_01_ = 1.000, *P*(*M|*data) = 0.496, providing the strongest support for the absence of any effects (see Table 3). The group effect (BF_01_ = 1.782) and the duration effect (BF_01_ = 15.147) yielded moderate to strong evidence for the null, and the interaction was decisively unsupported (BF_01_ = 57.259), providing very strong evidence against an interaction between duration and group. Thus autistic and non‐autistic adults rated trustworthiness in similar ways, with stimulus duration not affecting results either.

**TABLE 3:** Model comparison for all models under consideration for trustworthiness ratings.

Models	*P*(*M*)	*P*(*M*|data)	BF_M_	BF_01_	Error %
Null model	0.200	0.496	3.934	1.000	
Group	0.200	0.414	2.826	1.198	1.263
Duration	0.200	0.047	0.197	10.541	1.272
Duration + group	0.200	0.039	0.161	12.807	1.767
Duration + group + duration × group	0.200	0.004	0.017	114.066	4.221

*Note*: All models include subject and random slopes for all repeated measures factors.

Overall, the Bayesian analyses provided no strong evidence for differences in mean ratings of either age or trustworthiness between autistic and non‐autistic listeners across exposure durations. Importantly, these findings are consistent with previous work reporting no substantial differences between autistic and non‐autistic individuals in judging age and trustworthiness from faces (White et al., 2006).

### How much exposure do autistic and non‐autistic listeners need to form shared impressions?

We next investigated whether autistic and non‐autistic listeners differed from others in their own group in how quickly they formed impressions from voices at different exposure durations. To this end, we calculated the intraclass correlation coefficients (ICCs, ICC2k; two‐way random model, absolute agreement) and their 95% confidence intervals (CIs) for each characteristic and each exposure duration for each group using the *psych* package (version 2.4.6.26) in R (version 4.2.3). The ICC ranges from 0 (no agreement) to 1 (perfect agreement). A higher ICC indicates that listeners on average agreed on which voices sounded, for example, older vs. younger. Inter‐rater agreement can only arise when listeners have formed a shared impression from voices (Lavan, 2023; Mileva & Lavan, 2023). We inferred differences when the CIs around the ICCs did not overlap between the groups. While there is no direct mapping between CIs and *p*‐values, CIs that touch but do not overlap have been shown to be comparable to an *α* level of *p* = .01 (Cumming, 2009; Tan & Tan, 2010).

**FIGURE 3:**
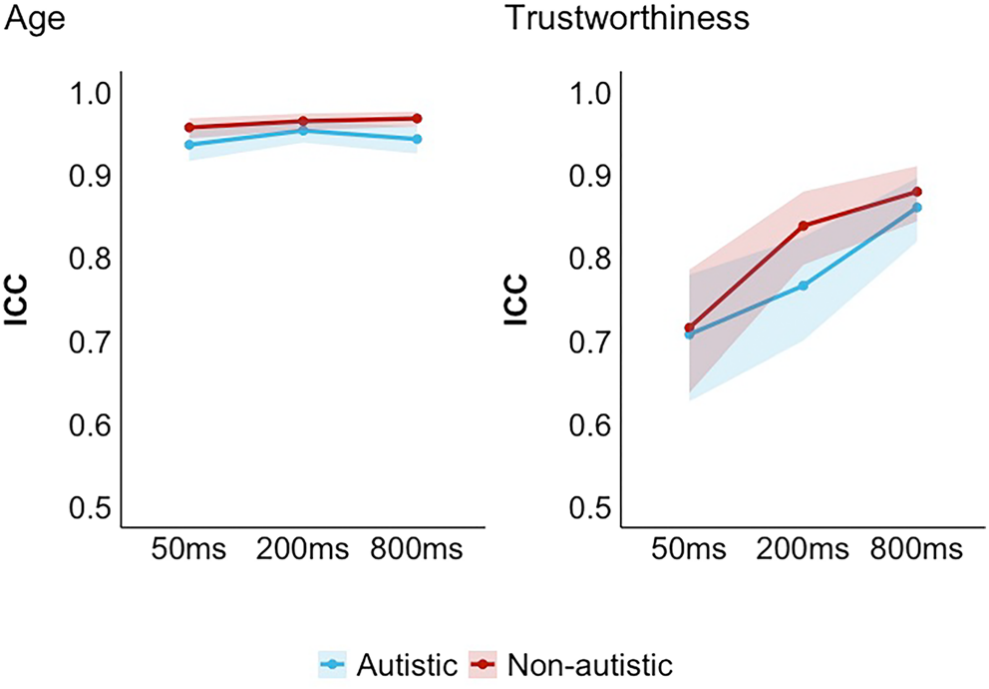
Intraclass correlation coefficients (ICCs) for ratings of both groups at each exposure duration, separately for Age (left) and Trustworthiness (right). Error bands represent 95% confidence intervals.

Impressions of age had overall high ICCs (at 50 ms: 0.94 for autistic and 0.96 for non‐autistic people; at 200 ms: 0.95 for autistic and 0.96 for non‐autistic people; at 800 ms: 0.94 by autistic and 0.97 by non‐autistic people) across all exposure durations in both groups (see Figure 3). We therefore observed a flat trajectory, where ICCs are high even after a very brief exposure, and ICCs do not increase substantially with longer exposure. This suggests that impressions of age are formed rapidly from voices, from even 50 ms of exposure to a voice, replicating Lavan's (2023) finding. Crucially, the CIs around ICCs overlapped between autistic and non‐autistic people for all exposure durations. This, therefore, suggests that first impressions of age are formed rapidly for both groups.

We found that first impressions of trustworthiness are formed somewhat more gradually than impressions of age, broadly replicating Mileva and Lavan (2023) and Lavan (2023). ICCs for trustworthiness increased from 50 ms of exposure (0.71 in the autistic group and 0.72 in the non‐autistic group) to 800 ms of exposure (0.86 in the autistic group and 0.88 in the non‐autistic group). Notably, however, ICCs were still relatively high throughout, across exposure durations and groups. As with age, impression formation for trustworthiness did not differ between autistic and non‐autistic listeners, with CIs overlapping across all exposure durations. These results therefore suggest that autistic and non‐autistic listeners can also form impressions of trustworthiness from relatively little exposure, but that having longer exposures increases agreement, suggesting that impressions evolve with increasing exposure.

### Are first impressions of autistic listeners more similar to those of other autistic listeners than to those of non‐autistic listeners?

**FIGURE 4:**
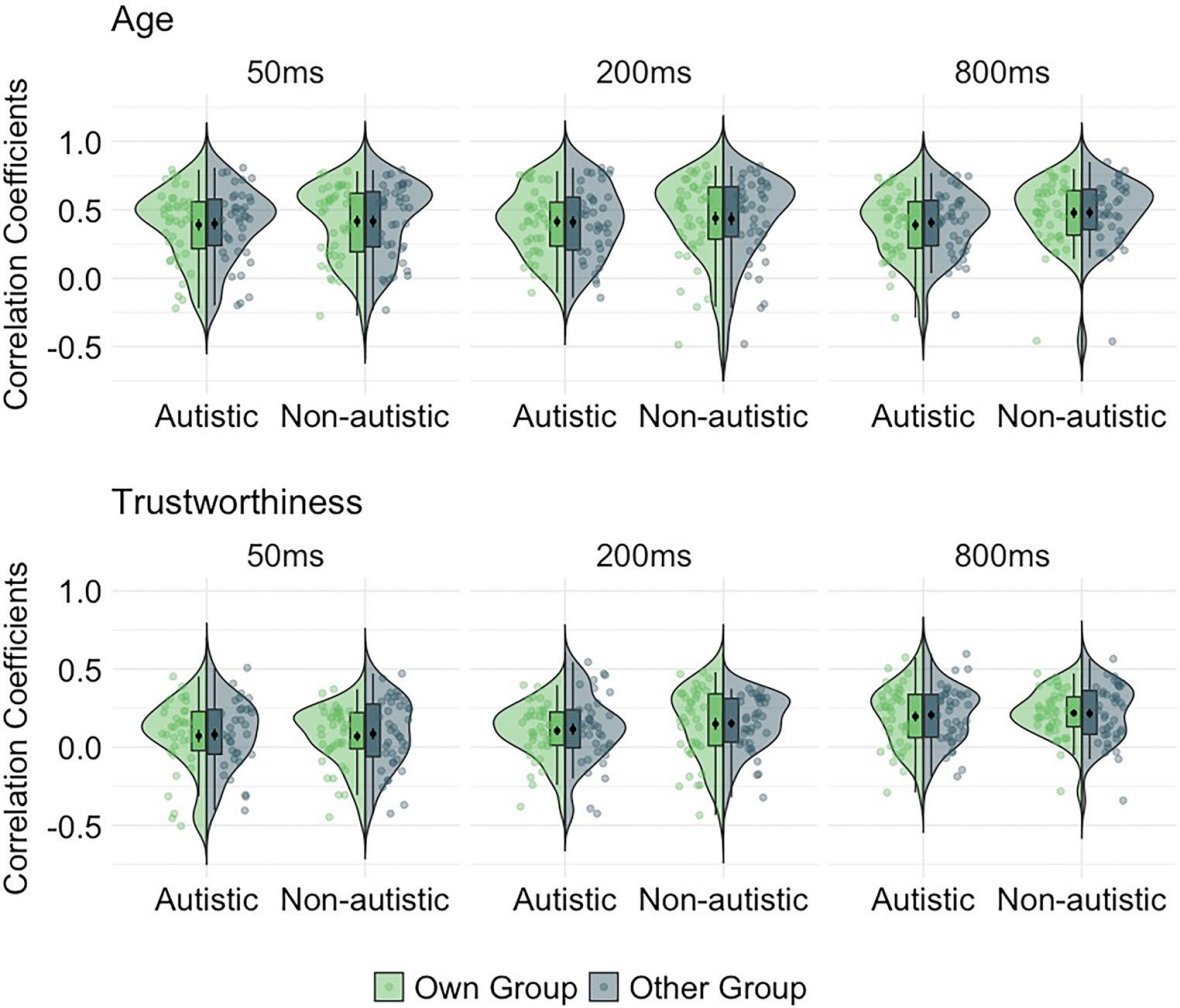
Own group and other group correlations for listeners at each exposure duration, separately for Age (above) and Trustworthiness (below). Black dot with short line inside the boxplot = mean ± 1 *SE*.

So far, we observed no substantial differences in how autistic and non‐autistic people form impressions from voices. We next examined whether more subtle differences between diagnostic groups might emerge by comparing agreement of ratings within and across groups; a recent theory of autism proposes that differences might only be seen across groups (Milton et al., 2022). Specifically, we correlated each participant's raw ratings with the mean ratings from their own group (leaving the participant's data out) and also with the mean ratings from the other group. This approach thus yielded two sets of correlation coefficients for each participant: ‘own group’ correlations and ‘other group’ correlations, which index how similar the ratings of each listener are to the ratings of their own group vs. the other listeners group. If there are any systematic differences between how autistic and non‐autistic listeners perceive person characteristics from voices, we should observe lower correlations between a participant's raw ratings and the other participant group (e.g. a non‐autistic listener's ratings correlated with mean ratings of the autistic listener group) than their own group (Figure 4).

We conducted a series of Bayesian paired‐samples *t*‐tests to examine differences between own‐group correlations and other‐group correlations, separately by characteristic (age, trustworthiness), exposure duration (50, 200 and 800 ms), and group (autistic and non‐autistic). As in previous analyses, BF_01_ > 1 indicates support for the null model, whereas BF_01_ < 1 indicates support for the alternative.

Results for non‐autistic listeners showed moderate to strong evidence in favour of the null hypothesis, indicating no meaningful difference between own‐ and other‐group correlations for both age and trustworthiness impressions. Results for autistic listeners also provided substantial to strong evidence in favour of the null hypothesis across most conditions, again suggesting no systematic differences between own‐ and other‐group correlations. Only for age impressions after 800 ms of exposure did we find moderate evidence in favour of the alternative hypothesis, suggesting a difference between own‐group and other‐group correlations. See Table 4.

**TABLE 4:** Bayesian paired‐samples *t*‐test results comparing own‐group and other‐group correlations.

Listener group	Characteristic	Duration	BF_01_	Posterior median *δ*	95% CI
Non‐autistic	Age	50 ms	5.846	−0.011	[−0.308, 0.286]
		200 ms	4.442	0.112	[−0.185, 0.413]
		800 ms	5.485	−0.05	[−0.352, 0.250]
	Trustworthiness	50 ms	3.906	−0.136	[−0.441, 0.166]
		200 ms	5.645	−0.026	[−0.331, 0.278]
		800 ms	5.749	0.03	[−0.267, 0.327]
Autistic	Age	50 ms	1.116	−0.284	[−0.598, 0.025]
		200 ms	5.315	0.063	[−0.237, 0.365]
		800 ms	0.145	−0.44	[−0.768, −0.119]
	Trustworthiness	50 ms	4.987	−0.068	[−0.390, 0.253]
		200 ms	4.948	−0.081	[−0.391, 0.227]
		800 ms	4.613	−0.103	[−0.406, 0.198]

*Note*: Posterior median *δ* and 95% CI refer to the posterior distribution of *δ*.

Overall, these findings suggest that autistic and non‐autistic adults form first impressions of age and trustworthiness from voices in very similar ways. The Bayesian analysis provided moderate to strong evidence supporting the absence of differences across most comparisons. The only exception was for age impressions at 800 ms in the autistic group, where we found moderate evidence for a difference between own‐ and other‐group correlations. However, this effect was small (as indicated by the corresponding posterior median *δ* and 95% CI [−0.768, −0.119]) and ran counter to theoretical expectations of greater within‐group similarity for autistic listeners. All analyses were conducted using JASP (version 0.19.3; JASP Team, 2025) with default priors (Jarosz & Wiley, 2014; van den Bergh et al., 2020).

## DISCUSSION

This is the first study to investigate whether autistic people differ from non‐autistic people in how they form first impressions from voices, and how quickly shared impressions are formed. Using a trait perception task with unfamiliar voices, participants rated an inferred characteristic (trustworthiness) and an apparent characteristic (age) of voices from 50, 200 and 800 ms of exposure, respectively. Using multiple analysis methods, we found that both non‐autistic and autistic listeners exhibited similar patterns of impression formation for age and trustworthiness across different exposure durations. In terms of the exposure required to form impressions, measured via inter‐rater agreement, autistic and non‐autistic listeners exhibited similar trajectories in impression formation over time from voices. In both groups, age impressions were well established across all time points, while impressions of trustworthiness gradually evolved from shorter to longer voice exposure durations, consistent with previous findings (Lavan, 2023). We further compared within‐ and across‐group correlation coefficients on agreement of ratings; autistic and non‐autistic listeners seem to form impressions of age and trustworthiness in very similar ways, indicating that autistic social differences do not always result in a lack of shared understanding (Milton et al., 2022).

The existing social perceptual literature on autism is predominantly focused on face processing, with relatively little research into voice processing, despite the crucial role of vocal communication in everyday social interactions. The speed and similarity with which autistic and non‐autistic listeners form first impressions highlight the shared nature of first impressions from voices: just like non‐autistic listeners, autistic listeners may extract subtle social information from the environment in order to develop impressions or may form impressions based on shared social stereotypes (Stolier et al., 2018). Our results contrast with the social difficulties often reported in previous literature in autistic people, suggesting that such challenges may be rather specific; in contrast, our study taps into a form of social ability in which autistic people's perception is indistinguishable from that of non‐autistic people, despite a history of clinically significant social communication difficulties associated with their diagnoses and their self‐reported high levels of autistic traits. Thus, this ability may be viewed as a possible social strength in autism. This suggests that the social challenges encountered by autistic people may be confined to specific areas of social perception rather than being universally pervasive. It is of course possible that challenges may arise for autistic people when faced with making trait judgments from more naturalistic or complex vocal stimuli and situations (Cai, Lavan, et al., 2024; Cai, White, et al., 2024). One limitation of the present study is the use of split‐second voices that lacked natural social context and meaningful interaction, which may have influenced how well the findings generalise to real‐world social cue interpretation. The nature of the stimuli may not fully capture the subtle differences in social perception between autistic and non‐autistic people. Future studies would benefit from using more ecologically valid stimuli that reflect complex, socially meaningful interactions. Additionally, they should aim to capture the full complexity and ecological validity of first impression formation in autism by exploring a wider range of traits. This would provide a deeper understanding of socially nuanced judgments and whether first impression formation is a potential relative social strength in autism.

Neuroimaging studies also hold the potential to shed light on the brain mechanisms involved in vocal trait impression production and perception in autistic people. Using fMRI to examine the expression of different vocal inferred social traits by the same speaker, non‐autistic people recruit the vocomotor network for basic vocal modulation, while social brain networks–including the medial prefrontal cortex, superior temporal sulcus, and precuneus–are specifically involved in social voice modulation (Guldner et al., 2020). Regarding the perception of social traits from voices, an EEG study has shown that first impressions of different traits from voices become more integrated over time in non‐autistic listeners (Lavan et al., 2024). Future studies could harness different neuroimaging methods to further explore first impression formation and production in autistic people, to reveal the underlying neural mechanisms through which autistic people extract and convey social information in voices, providing insights into the mechanisms underlying trait impressions that are independent of autistic social difficulties. Having a deeper understanding of first impression formation, in terms of whether and how impressions influence behaviour, would provide insight into the way in which vocal information shapes social interactions in autism.

## AUTHOR CONTRIBUTIONS

**Ceci Qing Cai:** Conceptualization; investigation; writing – original draft; methodology; writing – review and editing; formal analysis; project administration; data curation. **Rong Ma:** Investigation; project administration. **Terry Hin Ng:** Investigation; project administration. **Sarah J. White:** Investigation; funding acquisition; writing – original draft; methodology; writing – review and editing; supervision; conceptualization; visualization. **Nadine Lavan:** Conceptualization; investigation; funding acquisition; writing – original draft; methodology; writing – review and editing; visualization; formal analysis; supervision; resources.

## Data Availability

The data that support the findings of this study are available from the corresponding author upon reasonable request.
